# Primary care provider perceptions of enablers and barriers to following guideline-recommended laboratory tests to confirm chronic kidney disease: a qualitative descriptive study

**DOI:** 10.1186/s12875-018-0879-2

**Published:** 2018-12-10

**Authors:** Danielle M. Nash, Amit X. Garg, K. Scott Brimble, Maureen Markle-Reid

**Affiliations:** 1 ICES, London, Ontario Canada; 2 Department of Health Research Methods, Evidence, and Impact, McMaster University, Hamilton, Ontario Canada; 3 Department of Medicine, University of Western Ontario, London, Ontario Canada; 4 The Ontario Renal Network, Toronto, Ontario Canada; 5 Department of Medicine, McMaster University, Hamilton, Ontario Canada; 6 School of Nursing, McMaster University, Hamilton, Ontario Canada

**Keywords:** Chronic kidney disease, Laboratory tests, Qualitative research, Primary care, Family medicine, Theoretical domains framework

## Abstract

**Background:**

Patients should receive follow-up serum creatinine tests after an initial abnormal result to diagnose chronic kidney disease. However, half of the time this fails to occur in primary care. We interviewed primary care providers to better understand their perceptions of enablers and barriers to following this guideline-recommended care.

**Methods:**

We performed a qualitative descriptive study guided by the Theoretical Domains Framework (TDF), a framework for behavioural change. We used purposeful sampling to recruit primary care providers (physicians and nurse practitioners) based on provider and practice characteristics (rural, solo versus team practice, etc.) from Ontario, Canada. We completed one-on-one interviews with providers using a semi-structured and open-ended interview guide based on the 14 TDF domains. We alternated between data collection and analysis, where we used directed content analysis to identify frequent, important, and conflicting enablers and barriers.

**Results:**

We completed 13 interviews with nine primary care physicians and four nurse practitioners. Nine themes related to the TDF emerged from the data: 1) environmental context and resources, 2) knowledge, 3) memory, attention, and decision processes, 4) beliefs about consequences, 5) goals, 6) social or professional role, 7) behavioural regulation, 8) skills, and 9) optimism. Within these themes, we identified 16 enablers and five barriers. Some enablers included, providers’ knowledge on appropriate testing, their motivation to order these tests, and their use of tools and resources to help order follow-up serum creatinine tests. However, providers perceived some barriers including that ordering confirmatory laboratory tests for chronic kidney disease was not always a priority in regards to other care they wish to provide. Providers also noted that a perceived barrier is patients not going to the laboratory to complete the test.

**Conclusions:**

We identified novel enablers and barriers to primary care providers completing guideline recommended repeat testing for the diagnosis of chronic kidney disease. Similar research is needed to understand the views of patients. These research findings can be used to inform strategies to improve the quality of care.

**Electronic supplementary material:**

The online version of this article (10.1186/s12875-018-0879-2) contains supplementary material, which is available to authorized users.

## Background

Approximately 12.5% of all Canadians live with chronic kidney disease [1], which is characterized by a sustained reduction in kidney function and may include significant levels of protein in the urine. Early detection of chronic kidney disease allows healthcare providers to initiate appropriate management to help prevent or slow the patient’s progression to kidney failure. Most patients with early stage chronic kidney disease are managed in the primary care setting and are only referred to nephrologists if they have advanced disease or are at increased risk of progression.

International guidelines published in 2013 from Kidney Disease Improving Global Outcomes (KDIGO) recommend that chronic kidney disease should be classified based on estimated glomerular filtration rate (eGFR) and level of albuminuria [2]. These guidelines recommend that patients with an initial eGFR < 60 mL/min/1.73 m^2^ should have a repeat serum creatinine test within three months to diagnose chronic kidney disease [2]. Although these guidelines are well recognized by nephrologists, primary care providers are generally not aware of them. Additional efforts have been made in several jurisdictions to bridge this gap in primary care. For example, the Ontario Renal Network (ORN), the provincial agency responsible for the delivery of kidney care services in Ontario, Canada, released a flow diagram based on these clinical guidelines to aid primary care providers with appropriate screening, monitoring, management, and referral for chronic kidney disease (the KidneyWise toolkit) [3]. This toolkit provides specific advice for ordering follow-up serum creatinine tests. The ORN has attempted wide dissemination of this toolkit through national primary care conferences, social media, and integration into electronic medical records (EMRs).

Based on a previous population-based study among Ontario primary care providers, only 49% of patients with initial abnormal eGFR values received a repeat serum creatinine test in the following six months [4]. These findings are consistent with another Ontario study among primary care providers using an EMR [5]. Similarly, studies in other countries have shown that only 14 to 28% of patients with an initial eGFR < 60 mL/min/1.73 m^2^ have a documented diagnosis for chronic kidney disease [6–9]. It is not clear why this guideline recommendation is not being followed in practice for half of the patients.

Previous literature on evidence-practice gaps in primary care have demonstrated that guideline-based recommendations are generally not being followed in practice due to lack of time and resources, limited relevance of research to practice, and patient-related factors [10–16]. Based on our detailed literature review, we did not find any previous studies on primary care providers’ perspectives on the enablers and barriers to completing follow-up serum creatinine tests to confirm chronic kidney disease (Additional file 1). It is not clear if previously identified evidence-practice gaps are relevant to this practice.

There are many different frameworks and theories on clinical practice change and implementation of guidelines [17–19]. We used a robust framework of behavioural change, referred to as the Theoretical Domains Framework (TDF), in our study to shape our research questions, interview guide, and analysis [17, 20]. The TDF was developed to help understand why evidence-based guidelines may not be followed in practice and to help develop strategies to improve implementation of evidence into practice. It is a consensus framework based on 33 behaviour change theories and 128 theoretical constructs to inform implementation research [17]. Based on a validation study of the original TDF, the refined framework includes 14 domains and 84 theoretical constructs [20].

The purpose of this qualitative descriptive study was to use the TDF as a framework to elicit and describe the perceived enablers and barriers to following recommendations for ordering a repeat serum creatinine test after an initial abnormal kidney function test result by Ontario primary care providers.

## Methods

### Study design

We completed a qualitative descriptive study of primary care providers’ perceived enablers and barriers guided by the TDF. This study design was used to provide an in-depth description of enablers and barriers with minimal interpretation of the data [21–23]. We followed the reporting guidelines from the COnsolidated criteria for Reporting Qualitative Research (COREQ) (Additional file 2) [24].

### Ethics

In accordance with the Helsinki Declaration, we received research ethics approval from the Hamilton Integrated Research Ethics Board (# 2017–2286). We followed the Tri-Council Policy Statement 2 (TCPS2) guidelines on ethical conduct of qualitative research [25]. After sharing information about the study, we obtained verbal consent from providers for study participation. We sent the information and consent form by email to the participants if they preferred a copy. Participants received $25 compensation for their time, which was provided after each interview.

### Sampling & Recruitment

We used purposeful sampling strategies of maximum variation and snowball sampling to identify information-rich participants. Eligible participants included primary care providers practicing in Ontario who are responsible for ordering laboratory tests (physicians and nurse practitioners).

We used a multi-faceted recruitment strategy. First, we contacted the four main Ontario primary care organizations, which circulated information about our study to their members: 1) Association of Family Health Teams of Ontario, 2) Nurse Practitioners Association of Ontario, 3) Ontario College of Family Physicians, and 4) Association of Ontario Health Centres. These organizations provided our study information to their members either through a regular newsletter, by posting the information on their website, or through an online bulletin. The information provided included a brief explanation of the study and directed people to contact the research team for further information.

Second, we compiled a list of potential participants from the College of Physicians and Surgeons of Ontario and Nurse Practitioners’ Association of Ontario websites based on maximum variation selection criteria including rural versus urban clinics, provider sex, number of years practicing, family physician versus nurse practitioner, size of practice, and team versus individual practices. We contacted these individuals by calling or visiting primary care clinics in the province and briefly explaining the study to the healthcare providers or their administrative staff. We then provided practices with the study information flyer either by email, fax, or in person.

Third, we used snowball sampling by asking individuals to pass along our study information to potential participants to see if they would be interested in our study.

We decided a priori that we would recruit at least 13 participants. After the first 10 interviews, we planned on interviewing at least three more participants and completed recruitment when data saturation was reached and no new themes emerged with subsequent interviews [26, 27].

### Data collection

One author (DMN; Doctoral student) conducted all one-on-one, semi-structured, open-ended interviews either in person (at the physician office) or by telephone. We developed the interview guide based on the TDF and piloted it prior to the study with two physicians (see Additional file 3). We used the results of the pilot to modify and reframe questions. Example questions included, “How easy or difficult is it for you to regularly order confirmatory tests for chronic kidney disease?” and “What do you think will happen if you do not order confirmatory tests for chronic kidney disease?”. We revised questions slightly throughout the study as the data were analyzed. Based on the semi-structured format of the interviews, we used additional questions and probes to elicit further information or to ask participants to clarify answers. The interviews were audio recorded and transcribed verbatim. We uploaded the transcripts to NVivo 11 software for assistance with data management when performing the analysis.

We collected demographic and practice information, including provider’s age and number of years in practice, to provide some aggregate descriptive information on study participants.

### Analysis

We used directed content analysis by using the TDF as a framework to identify and describe the enablers and barriers derived from the data. Content analysis is a qualitative analytic technique where investigators systematically review and describe textual data using codes and themes [28]. Directed content analysis is both a deductive and an inductive analytic approach where an existing theory or framework is used to help guide the analysis and to generate initial concepts and themes [28]. Using this approach, we mapped the data to the 14 TDF constructs to help us identify enablers and barriers to guideline adherence for repeat serum creatinine testing to diagnose chronic kidney disease [17, 20]. We also identified any emerging themes from the data that did not fit into any of the TDF constructs.

The analysis was an iterative process, where we alternated between data collection and analysis. This allowed interview questions for subsequent data collection to be revised and also allowed us to determine when data saturation was reached.

We listened to the audio-recordings shortly after each interview and read through the transcripts to correct any errors in transcription and to fully immerse ourselves in the data.

The coding and broader themes were based on the TDF domains. We practiced investigator triangulation to increase credibility in research findings, where both DMN and MMR independently coded the first two interview transcripts, and compared coding to discuss agreement or disagreement. DMN completed the coding of the remaining transcripts independently, but with regular meetings with MMR to review coding progress. Any disagreements were resolved by consensus. We reviewed the transcripts from each interview and assigned initial codes based on the TDF domains to each data item, which typically included one to three sentences.

In the next phase of the analysis, we created sub themes within each broader domain. We then defined, refined and named these sub themes within the codebook, which was used to guide the remaining analysis, and revised throughout the study to reflect emerging themes. Finally, we identified the relevant TDF domains and sub themes by focussing only on the frequent, conflicting, or important themes (i.e. strong beliefs even if they were not as common across the participants).

Throughout the study, we practiced bracketing, which is the ability to separate our own values and opinions from influencing the participants’ responses or our interpretation of the results. This is especially important in qualitative research, since the findings are subjective and based on interpretation by the researchers. To ensure credibility and confirmability of findings, DMN kept a reflective journal throughout the research process to recognize, document, and try to separate any assumptions that may have influenced the research. To ensure credibility of research findings, we also used peer debriefing by meeting with other experienced qualitative researchers from the Ottawa Hospital Research Institute who are familiar with the TDF, but who were not involved in our study. We discussed and confirmed preliminary findings that emerged from the data after completing nine interviews. Finally, we kept a detailed audit trail including the initial study protocol, DMN’s reflective journal, audio recordings of the interviews, transcription files of the interviews, and minutes from research team meetings.

## Results

### Characteristics of the study participants

In total, we completed 13 interviews with 13 individual participants. We reached data saturation after the 10th interview, since no new themes emerged from interviews with participants 11 to 13. Nine out of 13 participants were female and the average age was 46 years. Nine participants were primary care physicians and the remaining four participants were nurse practitioners (see Table 1).

**Table 1:** Demographic and Practice Characteristics for the 13 Study Participants

Characteristics	Percentage/ Mean	Standard Deviation	Range (min – max)
Gender (% female)	69.2%		
Age (years)	45.8	9.2	29–59
Primary care physician or nurse practitioner (% primary care physician)	69.2%		
Number of years practicing	15.3	9.9	1–32
Medical school/ nurse practitioner program location (% Canada)	92.3%		
Practice location (% urban)	69.2%		
Practice type (% family health team/ family health group)	92.3%		
Approximate number of patients rostered/ in the practice	2248	3219	200–12,500

### Relevant TDF domains

Themes emerging from the data reflected nine of the TDF domains: 1) environmental context and resources, 2) knowledge, 3) memory, attention and decision processes, 4) beliefs about consequences, 5) goals, 6) social or professional role, 7) behavioural regulation, 8) skills, and 9) optimism (see Table 2). In addition to these TDF themes, another theme emerged on completing laboratory tests/ patient factors.

**Table 2:** Relevant Theoretical Domains Framework (TDF) Themes and Sub-themes Identified as Enablers or Barriers

Domain/ Theme	Sub-theme	Relevance	Enabler/ Barrier
Environmental context and resources	Using EMR tools	Frequent	Enabler
	Referring to guidelines	Frequent	Enabler
	Depending on support staff	Frequent	Enabler
Knowledge	Being aware of guidelines	Conflicting	Enabler
	Having a positive attitude toward guidelines	Conflicting	Enabler & Barrier
	Knowing what to do	Frequent	Enabler
Memory, attention and decision processes	Making a deliberate decision	Frequent	Enabler
	Forgetting	Important	Barrier
Beliefs about consequences	Being aware of clinical consequences	Frequent	Enabler
	Perceived low risk in delaying confirmatory test	Important	Barrier
	Weighing the costs and benefits	Frequent	Enabler
Goals	Prioritizing care goals	Conflicting	Enabler & Barrier
	Recognizing the importance	Frequent	Enabler
Social or professional role	Claiming responsibility	Frequent	Enabler
	Identifying practice type or role influences	Frequent	Enabler & Barrier
Behavioural regulation	Taking ownership of action	Frequent	Enabler
Skills	Demonstrating communication skills	Important	Enabler
Optimism	Having a positive attitude	Frequent	Enabler
N/A^a^	Completing laboratory tests/ patient factors	Frequent	Barrier

^a^This was not one of the TDF domains but was considered a relevant theme

### Identified TDF enablers

Among the nine TDF themes, we identified 16 enablers perceived by primary care providers to ordering repeat serum creatinine tests (see Table 2). The majority of providers were aware of guidelines for ordering a repeat serum creatinine test and most had a positive opinion about using clinical guidelines to inform behaviour and decision-making (*knowledge*). For example: *“I don’t think [guidelines] should determine [behaviour], but they should definitely guide it and direct it because it’s, again, research based and trying to follow that.”* The most commonly used clinical guidelines were for diabetes or the implementation of KDIGO chronic kidney disease guidelines in the ORN’s KidneyWise toolkit (*environmental context and resources*). For example:

*“I think that the algorithm approach is actually relatively simple as opposed to a lot of the other guidelines out there that have algorithms that are about three hundred things on a diagram and then having an application for it is useful. The KidneyWise application is actually quite useful.”*

Although not all providers were aware of guidelines for ordering repeat serum creatinine tests, the majority still had the knowledge of when they should be ordering these tests (*knowledge*). For example:

*“Let’s just say I don’t know anything about any guidelines. I have a practice that I do, that I believe is correct, so we’ll see what happens there.”* and *“Actually there’s one today that just popped up that his glomerular filtration rate dropped like from 70 to 50 which is below normal, so I’m going to repeat it in three months.”*

Furthermore, providers described that they would refer to guidelines when needed and then tailor the recommendations to the specific patient and their clinical presentation in order to decide when they should order confirmatory tests for chronic kidney disease (*environmental context and resources; memory, attention and decision processes*). For example:

*“So usually the first thing if I get an abnormal creatinine or estimated glomerular filtration rate or positive albumin-to-creatinine ratio then it’s to, kind of, look and see, okay, is this something new for this person or is this long-standing, is it getting worse, is it stable, is there something else going on, do they have a urinary tract infection… like, something that may account for the finding. If it’s something that’s completely new then, absolutely, it’s repeated.”*

Besides using clinical guidelines, providers frequently described the use of internal clinic resources to help decide when or if to order a repeat serum creatinine test (*environmental context and resources*). Many providers described the use of support staff (i.e. clerical staff or nurses) to follow up with patients about a repeat serum creatinine test. For example: *“I can just send tasks to certain nurses or support staff just to follow back up with them and ask them to order whatever I need to be done.”* Even though providers agreed that having support staff would be helpful, not all providers had available support staff to assist with ordering laboratory tests or to follow-up with patients (*environmental context and resources*). For example:

*“If the world was a perfect place some of this stuff could be off loaded to either a nurse or a nurse practitioner that I work with but the world is not a perfect place and we’re all just too busy.”*

Providers frequently described using different features in their EMRs to help decide whether or not to order laboratory tests or to remind themselves to order a follow-up test (*environmental context and resources; memory, attention and decision processes*). For example: *“The electronic medical record that allows me to kind of track… laboratory results of creatinines over time, is something that helps me determine whether or not I need to do a confirmatory test.”* A couple providers also mentioned that having chronic disease registries (mostly for diabetes) could be used to help keep track of patients who may require a follow-up serum creatinine test. However, they mentioned that these registries generally require the help of support staff (who may or may not be available) to manage and track when patients need certain laboratory tests.

Many providers agreed that ordering a repeat serum creatinine test is a priority and helps to prevent potential adverse consequences for the patients (*goals*; *beliefs about consequences*). For example:

*“You’ve just got to focus in on one or two different things, and sometimes the chronic kidney disease could get lost in transition. But usually it’s incorporated, but that would be the most likely.”* and *“One [consequence] is that it continues to go up, and I miss that they’re going into much worse renal failure. Another is that I give them things that are more toxic, or that are toxic to an already compromised kidney. Those would be the biggest ones.”*

Providers also agreed that the benefits of ordering these tests outweigh the costs to the healthcare system (*beliefs about consequences*). For example: *“Yes, because it’ll cost a lot more if [their kidney function] declines because we didn’t check it.”*

Overall, providers were generally optimistic about ordering follow-up laboratory tests for chronic kidney disease and were motivated to do so (*optimism; goals*). For example: “*Like there are no concerns about ordering any of these tests.”* and *“… I try very hard, because I mean, kidneys are pretty important, right?”*

All participants agreed that ordering a follow-up serum creatinine test to confirm chronic kidney disease is part of their role as primary care providers (*social or professional role*). For example: *“Physicians have to be the ones in Ontario signing blood work requisitions, nurse practitioners and physicians.”* Some participants described components of their professional role that enable them to order a repeat serum creatinine test. For example:

*“As a nurse practitioner I’m allowed a little bit more time so it makes it a little easier, so I try and provide as much health teaching to the patient and write it on the lab slip when I want them to check it.”*

### Identified TDF barriers

We identified five barriers perceived by primary care providers to ordering a repeat serum creatinine test to diagnose chronic kidney disease (see Table 2). There were some conflicting perspectives on views of clinical guidelines where some providers had more pessimistic views (*knowledge*). For example:

*“I’m going to assume that [guidelines] are evidence based or at least partially evidence based as much as guidelines can be because if you look at those guidelines in general they’re about maximally 14% evidence based and the rest is opinion, so I assume that they are approximately the same as every other guideline.”*

Some providers did not perceive that it was a priority to order a repeat serum creatinine test relative to other competing priorities in primary care (*goals*). For example:

*“So I’ll tell you what, we have 49 diseases that we deal with in family medicine. Kidneys are one small one, and there’s very little to do with that repeat creatinine. There’s nothing that changes. So is it a priority? No. There are many other things that are higher priority.”*

Providers also described that sometimes they forget to order the repeat testing (*memory, attention and decision processes*), but many mentioned that using the EMR as a resource generally helps to prevent forgetting (*environmental context and resources*). For example:

*“I think cognitive overload probably plays a part in everything that we do every day and it’s a matter of sometimes things just get forgotten.”* and *“…I guess once upon a time for me it would have been remembering when it was due. But electronic medical records make it that much easier because you can send yourself little reminders.”*

Even though the majority of providers agreed that there are significant clinical consequences for the patients if follow-up laboratory tests are not ordered, a few providers perceived that waiting longer to confirm the initial test result would not change the care they provide for the patient (*beliefs about consequences*). For example:

*“You know, it’s nice to initiate in the workup once they are confirmed [chronic kidney disease] a little bit earlier, but if it has to wait until a year, I don’t know that it makes a significant difference, ‘cause patients usually present on an annual basis for blood work. Or that’s their expectation. So sometimes you only have the chance to repeat it a year later.”*

Some providers described components of their professional role that prohibit them from always ordering follow-up laboratory tests (*social or professional role*). For example, providers who work in a family health team described the following: *“…because they’re multiple providers… it may be something that someone else has already investigated.”*

### Other factors influencing laboratory test completion

The providers in our study had perceived some patient barriers to completing repeat serum creatinine testing that did not fit within any of the TDF domains. For example, the most prevalent barrier identified was patient compliance (*completing laboratory tests/ patient factors*). Some providers described using communication skills with the patients to explain the importance of getting the laboratory test done, which they perceived helps to improve patient compliance (*skills*). For example: *“So, you know, we usually tell them that, no, we need to repeat this because your renal function, we need to make sure your kidneys are good. And then … they’re on board.”*

In addition, providers described other actions that they take to help improve patient compliance in completing laboratory tests when ordered (*behavioural regulation*). For example:

*“Providing more follow-up and making sure, again, tests are being done as asked of the patient just to make sure they are. So having maybe more tasks sent to myself reminding myself that things have been ordered, to recheck that.”* and *“Well, one thing that I will tell you is that I do not file the abnormal test into the patient’s chart until I am sure that the patient actually is aware of the abnormal result.”*

Providers also mention laboratory factors which may influence whether or not the test is ordered, and ultimately if the patients complete the test (*completing laboratory tests/ patient factors*). For example: *“We used to have a lab in our family practice unit, right in the same building and that really was helpful for our patients in terms of any sort of laboratory investigations, but yeah.”*

## Discussion

Using a comprehensive framework of behaviour change to guide our analysis, we identified 16 enablers and five barriers perceived by providers for ordering repeat serum creatinine tests to diagnose chronic kidney disease. We found that there was an interaction between many of the TDF domains. For example, healthcare providers generally know what they should be doing (*knowledge*), are motivated to do so (*goals*), have the tools and resources required to perform the behaviour (*environmental context and resources*), and use both the information and tools to make an informed decision on whether or not to order a repeat serum creatinine test (*memory, attention and decision processes*). However, ordering follow-up serum creatinine tests was not always perceived as a priority (*goals*) or as directly influencing patient outcomes (*beliefs about consequences*), and might sometimes be forgotten (*memory, attention and decision processes*)*.*

Based on our comprehensive literature search, this is the first qualitative study to assess the enablers and barriers perceived by primary care providers for the behaviour of ordering repeat serum creatinine tests to confirm a chronic kidney disease diagnosis. We found novel enablers and barriers that have not been reported in previous studies related to chronic kidney disease care or laboratory ordering in general, including the following enablers: making a deliberate decision and being aware of clinical consequences; and barriers: forgetting to order tests and prioritizing care goals.

Unlike other studies on guideline adherence for chronic kidney disease care, we found that providers are generally aware of guidelines or at least know that they should be ordering repeat serum creatinine tests to confirm chronic kidney disease [29–32]. Previous studies have shown low awareness of national (U.S.) and international guidelines specifically for chronic kidney disease. In contrast, we were interested in participants’ awareness of any guidelines for confirming chronic kidney disease with repeat serum creatinine testing [2, 33]. As such, our participants were generally more aware of diabetes guidelines and a provincial kidney algorithm.

Although the majority of participants in our study had positive attitudes towards clinical guidelines for ordering follow-up serum creatinine tests, there were some dissenting views perceiving that these guidelines lacked clinical evidence. Estrella et al. (2003) conducted a survey with healthcare providers and also found that many participants did not perceive chronic kidney disease guidelines to be evidence-based [34].

Consistent with our study findings, previous studies have found that primary care providers perceive laboratory tests to be useful in assessing kidney function [31, 35]. However, we found that even though providers were generally motivated to order a repeat serum creatinine test and perceived this to be important, it was not always a priority. Furthermore, we identified another barrier where some participants perceived that the care they provide for the patient will not change if they do not order follow-up serum creatinine tests in a timely manner. This result is consistent with findings from Crinson et al. (2010), who performed focus groups with primary care providers on their perceptions of chronic kidney disease management [35].

Similar to our findings, previous studies have also shown that the use of internal resources are enablers to caring for patients with chronic kidney disease and ordering laboratory tests. For example, providers generally rely on support staff and use electronic medical records to provide better patient care [36–39].

Finally, previous studies on primary care providers’ perceived enablers and barriers to ordering laboratory tests in general have also identified that a barrier is patients not completing the laboratory test [37, 40]. A mixed methods study on patient perceived barriers to not completing a laboratory test after initiating a new medication that required monitoring included barriers of forgetting or competing demands [41]. Since our study only included the primary care provider perspective, we cannot make any conclusions about the applicability of these patient-specified barriers to completing a repeat serum creatinine test. Additional research is needed to further investigate these findings.

### Strengths and limitations

We used strategies suggested by Guba (1981) as outlined in our methods section to ensure rigour in our qualitative study [42]. This helps to increase the credibility, dependability, confirmability, and transferability of our study findings.

Another strength of our study is using the TDF to help frame our research, since it is comprehensive, validated, and has been successfully used in previous research on guideline implementation [17, 20]. Furthermore, the TDF also includes clearly defined domains that were applicable to our setting. A limitation of using a framework to guide our study is that we may have missed themes that were not captured through the TDF.

The findings from our study are transferable to other settings, for instance, primary care providers who work in similar primary healthcare settings across Canada. Based on the maximum variation sampling criteria that we applied, and our multi-faceted recruitment strategies, our findings likely apply to primary care physicians and nurse practitioners, female and male providers, urban and rural clinic settings (including Northern Ontario), practices of varying sizes, different types of practice models, and providers who have been practicing for different lengths of time. Even though we included both urban and rural primary care providers in our study, the majority of rural providers were nurse practitioners. This may have limited the perception specifically of rural primary care physicians. Furthermore, the majority of the participants in our study practiced in a family health team or a family health organization, thereby limiting the perceptions captured by solo-practicing providers. Previous literature has shown that providers perceive lack of effective reminders or tools to track laboratory tests in the EMRs is a barrier to ordering tests [37–39]. All participants in our study described that they used an EMR to order tests and most described this as an enabler. Therefore, our findings may not apply to primary care providers who do not use an EMR in practice.

### Study implications

This research has implications on the care of patients with chronic kidney disease in the primary care setting. The results of our study can be used to inform future interventions to help improve care regarding repeat serum creatinine tests to diagnose chronic kidney disease.

Future strategies to improve confirmatory laboratory test ordering for chronic kidney disease need to be multi-factorial since many components of the TDF apply to this behaviour. By mapping the relevant TDF domains to the Behaviour Change Wheel, we can identify interventions that may help to improve adherence to this behaviour [20, 43]. For example, since we showed that an enabler is use of tools within EMRs, we could use an environmental restructuring intervention to improve reminders or prompts within the EMRs to order follow-up laboratory tests. As another example, we could use a persuasion intervention such as presenting convincing yet factual information on the importance of ordering confirmatory tests to delay disease progression. This would help overcome the perceived barriers of ordering repeat serum creatinine tests being a low priority and that there is little harm in delaying confirmatory testing.

The findings from this research can potentially be generalized to guideline-recommendations in primary care beyond chronic kidney disease. For instance, some of the enablers and barriers identified in this study might be applicable to confirmatory testing for hypothyroidism [44]. However, future research is needed to explore the applicability of these factors to the implementation of other guidelines in primary care.

## Conclusions

Overall, we identified some novel enablers and barriers perceived by primary care providers in regards to ordering repeat serum creatinine tests to diagnose chronic kidney disease. The majority of participants know that they should be ordering these tests, and are generally motivated, and have the required resources to do so. However, some providers perceived that ordering a repeat serum creatinine test would not change the care they provide and it may not always be a priority to the provider or the patient. Providers also perceived that there may be other contributing factors beyond their control, such as patients not going to the laboratory to complete these tests. Future qualitative research with patients as the participants is needed to confirm and further investigate this finding.

## Additional files

Additional file 1: Literature review of previous relevant qualitative research studies. (DOCX 32 kb)

Additional file 2: Consolidated criteria for reporting qualitative research (COREQ). (DOCX 19 kb)

Additional file 3: Interview Guide Questions. (DOCX 26 kb)

## Abbreviations

eGFR: Estimated Glomerular Filtration Rate
EMR: Electronic Medical Record
KDIGO: Kidney Disease Improving Global Outcomes
ORN: Ontario Renal Network
TCPS2: Tri-Council Policy Statement 2
TDF: Theoretical Domains Framework
