# Supplementary material for: Primary care provider perceptions of enablers and barriers to following guideline-recommended laboratory tests to confirm chronic kidney disease: a qualitative descriptive study
Source: BMC Fam Pract. 2018 Dec 10;19:192. doi: 10.1186/s12875-018-0879-2 (PMC6287355; doi:10.1186/s12875-018-0879-2)
Supplement: Supplementary file 1 — Literature review of previous relevant qualitative research studies. (DOCX 32 kb) [file 12875_2018_879_MOESM1_ESM.docx]

**Additional file 1: Literature review of previous relevant qualitative research studies**

| **Reference** | **Study Objective** | **Brief Methodology** | **Summary of Results** |
| --- | --- | --- | --- |
| **Qualitative Research on Implementing Guidelines for Chronic Kidney Disease in Primary Care** | | | |
| Lo et al., 2016 [1] | To explore how the care for patients with CKD and diabetes can be improved according to primary and tertiary care providers. | Qualitative study, which included a combination of focus groups with care providers and interviews with the unit heads. | Sixty-five primary and tertiary care providers participated. Important factors included: lack of patient self-management, poor access to tertiary care, poor coordination and integration across primary and tertiary care, reactive approach to care where focus is on treatment rather than prevention, and need for more participation in quality improvement activities. |
| Vest et al., 2015 [2] | To understand barriers to guideline implementation for patients with CKD. | Mixed methods study of a cluster randomized trial with an embedded qualitative component. Semi-structured interviews with physicians. | Themes were organized into the components of the Normalization Process Theory: coherence (some physicians not aware of guidelines – or some of the aspects – for CKD, mostly used guidelines to help diagnose, lack of continuing education and challenge of keeping up with changing guidelines, some physicians do not like to give their patients the label of CKD or rather refer to the nephrologist to diagnose), cognitive participation (challenges with population health management and keeping up with all the different initiatives; challenge of unmotivated or non-adherent patients), collective action (limited time and resources/ staff, and competing demands) and reflexive monitoring (do not currently receive audit and feedback reports for CKD, data collection and management challenging and time consuming). |
| Blakeman et al., 2012 [3] | To identify processes underpinning the implementation of CKD management in primary care. | Qualitative study following the Normalization Process Theory. Interviews with PCPs and nurses from 19 participating sites from another study. | Three main themes emerged all around the anxiety associated with disclosure of CKD with patients: 1) tensions related to identifying and discussing CKD in older patients or those with early CKD, 2) embedding early-stage CKD within vascular care, and 3) distribution of work within the practice team. These research findings suggested that the current approach to management of early-stage CKD in primary care may miss opportunities to address susceptibility to renal damage, improve self-management of cardiac conditions, and improve the management of multi-morbidity. |
| Greer et al., 2012 [4] | To identify PCPs’ perceived barriers of educating patients about CKD. | Qualitative study using focus groups with PCPs. Analysis used methods from grounded theory. | Eighteen PCPs participated in 3 focus groups. Six main barriers emerged: 1) Patients not aware of CKD or not recognizing it as a medical problem, 2) PCPs not perceiving CKD as a distinct medical condition, 3) PCPs lack of knowledge to properly educate patients, 4) Do not want to overwhelm patients with a new diagnosis, 5) Time constraints with patients (no time for education), and 6) Lack of available educational resources. |
| Crinson et al., 2010 [5] | To assess the perspectives of PCPs on the new UK guidelines for CKD. | Qualitative diagnostic analysis using focus groups to inform the intervention of a clinical trial. Purposive sampling of 5 of the 70 practices that had agreed to participate in the clinical trial. | Eight themes emerged:  1. General responses to CKD  2. Issues surrounding use of the eGFR measures  3. Labeling issues: belief that kidney disease part of normal aging process  4. Issues surrounding giving a CKD diagnosis  5. Issues surrounding the management of blood pressure in CKD  6. Patient self-management and compliance issues in relation to meeting blood pressure targets  7. Nephrology referral issues  8. Educational requirements of practice regarding CKD |
| Feldstein et al., 2010 [6] | To understand the barriers and facilitators to care for CKD in primary care. | Qualitative study which included initial interviews to develop broad questions to be used for focus groups with PCPs and nurse practitioners. | There were 26 participating care providers in 5 focus groups. Barriers and facilitators included: guideline and patient factors (guidelines not flexible, patients not self-managing, patient costs and dealing with multiple providers), PCP factors (lack of time, lack of knowledge and attitudes about guidelines), system factors (lack of decision support tools, low physician autonomy, poor access to information, systems made for acute not chronic diseases). Facilitators included clear and specific guidelines, awareness of guidelines, clinical decision support tools, clinic support staff and easy access to patient information. |
| Williams et al., 2008 [7] | To determine factors associated with adherence to multiple medications for patients with CKD and diabetes. | Descriptive exploratory qualitative study including structured interviews with patients who have CKD and diabetes and focus groups with physicians. | Results included themes of: 1) purposeful action (patients did not have a strong intention to take medications, which went against what physicians thought); 2) perceived need (patients did not think they needed all medications or know why they were taking them); 3) perceived effectiveness (patients did not perceive all their medications to be effective); 4) medication safety (medications causing side effects according to patients, physicians think they may be over-reacting in most cases); 5) access (patients who forgot to refill prescriptions, although pharmacists helped prevent this; other access barriers of costs, lack of transportation, physical symptoms); 6) routine (patients disliked change to their medication routines); 7) remembering (patients forgetting to take medications; physicians noted that poor mental health was a barrier); 8) feedback (physician-patient relationship was important to adherence; patients lacked knowledge about what medications were for; some patients used prompts to remember to take pills). |
| Fox et al., 2006 [8] | To assess PCPs’ knowledge of CKD and uptake of Kidney Disease Outcome Quality Initiative (K/DOQI) guidelines. | Qualitative study using semi-structured interviews and surveys with PCPs. | Ten PCPs participated in the study. Five main themes emerged: 1) lack of awareness of K/DOQI guidelines (none of physicians reported being aware of or using guidelines), 2) suggestions for more CKD guidelines (strongly believed in following guidelines, especially for hypertension and diabetes but not for CKD), 3) use of outdated approaches (using serum creatinine rather than eGFR and proteinuria to diagnosis and monitor), 4) inconsistent methods to treat comorbidities, and 5) uncertainty about timing for nephrology referral (have own rules on when to refer and most reported difficulty in communicating with nephrologists and sharing care of patient). |
| **Research on Reasons for Care Gaps Regarding Laboratory Test Ordering (non-Chronic Kidney Disease)** | | | |
| Litchfield et al., 2014 [9] | To understand PCPs’ reasons for ordering liver function laboratory tests. | Prospective study with interviews. | Factors categorized into:  1) Internal: general attitudes and efficacy of ordering liver function tests and  2) External: social influence and test characteristics. This may take precedence over internal/ clinical characteristics. |
| Menon et al., 2014 [10] | To identify factors associated with facilities who had low or high missed test results in the Veterans Affairs electronic medical record system. | Mixed-methods evaluation, which used previous surveys to classify facilities as low or high risk and qualitative interviews with these facilities. | Forty facilities participated (20 low risk and 20 high risk). High risk scenarios for missed tests included: tests ordered by trainees were sometimes missed if laboratories only notified the ordering physician and not an attending physician as well; assignment of surrogates when physicians were on extended vacation/ leave where surrogates were not always assigned or did not always act on the test result; patients may not have been assigned to a PCP in the electronic medical record system. |
| West et al., 2014 [11] | To understand the perceived care gaps for laboratory management in practice. | Survey to clinicians and staff in Colorado primary care practices. | There were 384 completed surveys from 21 practices. Barriers included human error and communication issues during handoff, difficulty with sorting through laboratory results, patient no shows to laboratories, and outdated contact information when trying to notify patients. |
| Goldman et al., 2010 [12] | To understand physicians’ perspectives on laboratory monitoring for drugs. | Qualitative study using focus groups with physicians. | Twenty-nine physicians participated (20 internal medicine physicians or PCPs and 9 specialists). The participants agreed that laboratory monitoring was an important and time-consuming (and non-reimbursed) part of their practice. They were surprised by the number of errors that had been reported in the literature but recognized that errors sometimes do occur. Barriers included: uncertainty about who is responsible for ordering a laboratory test (i.e. when specialist prescribes it); uncertainty about when to order laboratory tests (i.e. for drugs perceived as low-risk); absence of automated reminders to keep track of laboratory test ordering; patients not completing laboratory tests that were ordered. A facilitator included workflow procedures (i.e. patients coming into the office each month to receive new prescription) and being able to tailor electronic medical record alerts for their practice (e.g., certain follow-up intervals for drugs). There were some concerns about alert fatigue or the number of clicks required to deal with alerts. |
| Elder et al., 2009 [13] | To understand laboratory test result management systems in family physician offices and to understand factors for optimal management. | Multi-method study using observations, interviews and surveys among 4 purposefully selected family medicine clinics. | Some themes that emerged included:  1) Safety awareness (leadership communicates and demonstrates commitment to safety and quality care; communication between staff and physicians on safety and quality care; teamwork; procedures and protocols for managing tests exist and are kept up-to-date);  2) Adoption of technology (electronic medical records incorporate office management tasks; digital ordering of laboratory tests and for receiving results; electronic medical records generate communication of laboratory results to patients; return of laboratory results is an automated step in the electronic medical records). Having technology was not a prerequisite for better laboratory management, rather having safety awareness and good communication in sites without electronic medical record technology still showed optimal management strategies. |
| Parker et al., 2008 [14] | To understand barriers and enablers to adhering to the national cholesterol guidelines in Rhode Island. | Qualitative study including 9 focus groups with physicians. | Summarized results only focus on enablers and barriers to laboratory tests from this study.  Fifty primary care physicians participated. Barriers included reimbursement issues for cholesterol screening and management and lack of reminder systems and the cost of informing patients about their laboratory results. Enablers included technologies to rapidly test for cholesterol levels in practice and reminders with point of care guidelines through electronic medical records to assess cholesterol levels. |

**References**

1. Lo C, Ilic D, Teede H, Cass A, Fulcher G, Gallagher M, et al. The Perspectives of Patients on Health-Care for Co-Morbid Diabetes and Chronic Kidney Disease: A Qualitative Study. PLoS One. 2016;11:e0146615.

2. Vest BM, York TRM, Sand J, Fox CH, Kahn LS. Chronic Kidney Disease Guideline Implementation in Primary Care: A Qualitative Report from the TRANSLATE CKD Study. J. Am. Board Fam. Med. 2015;28:624–31.

3. Blakeman T, Protheroe J, Chew-graham C, Rogers A, Kennedy A. Understanding the management of early-stage chronic kidney disease in primary care : 2012;233–42.

4. Greer RC, Crews DC, Boulware LE. Challenges perceived by primary care providers to educating patients about chronic kidney disease. J. Ren. Care. 2012;38:174–81.

5. Crinson I, Gallagher H, Thomas N, de Lusignan S. How ready is general practice to improve quality in chronic kidney disease? A diagnostic analysis. Br. J. Gen. Pract. 2010;60:403–9.

6. Feldstein D, Baier Manwell L, Grasmick M. Primary Care Providers and Chronic Kidney Disease Management: a Qualitative Study. Soc. Gen. Intern. Med. 2010;33:S368.

7. Williams AF, Manias E, Walker R. Adherence to multiple, prescribed medications in diabetic kidney disease: A qualitative study of consumers’ and health professionals’ perspectives. Int. J. Nurs. Stud. 2008;45:1742–56.

8. Fox CH, Brooks A, Zayas LE, McClellan W, Murray B. Primary care physicians’ knowledge and practice patterns in the treatment of chronic kidney disease: an Upstate New York Practice-based Research Network (UNYNET) study. J. Am. Board Fam. Med. 2006;19:54–61.

9. Litchfield I, Lilford R, Bentham L, Greenfield S. A qualitative exploration of the motives behind the decision to order a liver function test in primary care. Qual Prim Care. 2014;22:201–10.

10. Menon S, Smith M, Sittig D, Petersen N, Hysong S, Espadas D, et al. How context affects electronic health record-based test result follow-up: a mixed-methods evaluation. BMJ Open. 2014;4:e005985.

11. West DR, James KA, Fernald DH, Zelie C, Smith ML, Raab SS. Laboratory Medicine Handoff Gaps Experienced by Primary Care Practices : A Report from the Shared Networks of Collaborative Ambulatory Practices and Partners ( SNOCAP ). J Am Board Fam Med. 2014;27:796–803.

12. Goldman RE, Soran CS, Hayward GL, Simon SR. Doctors’ perceptions of laboratory monitoring in office practice. J. Eval. Clin. Pract. 2010;16:1136–41.

13. Elder N, McEven T, Flach J, Gallimore J. Management of Test Results in Family Medicine Offi ces. Ann Fam. Med. 2009;343–51.

14. Parker DR, Gramling R, Goldman RE, Eaton CB, Ahern D, Cover RT, et al. Physicians’ Perceptions of Barriers and Facilitators Regarding Adoption of the National Cholesterol Education Program Guidelines. Prev. Cardiol. 2008;Winter:29–35.
