# Supplementary material for: Primary care provider perceptions of enablers and barriers to following guideline-recommended laboratory tests to confirm chronic kidney disease: a qualitative descriptive study
Source: BMC Fam Pract. 2018 Dec 10;19:192. doi: 10.1186/s12875-018-0879-2 (PMC6287355; doi:10.1186/s12875-018-0879-2)
Supplement: Supplementary file 2 — Consolidated criteria for reporting qualitative research (COREQ). (DOCX 19 kb) [file 12875_2018_879_MOESM2_ESM.docx]

**Additional file 2: Consolidated criteria for reporting qualitative research (COREQ)**

| **Guide description** | **Guide questions** | **Where reported/ Answers** |
| --- | --- | --- |
| **Domain 1: Research team and reflexivity** | | |
| Personal characteristics | | |
| 1. Interviewer/ facilitator | Which author/s conducted the interview or focus group? | Data Collection |
| 2. Credentials | What were the researcher’s credentials? E.g. PhD, MD | Data Collection |
| 3. Occupation | What was their occupation at the time of the study? | Data Collection; Authors’ Information |
| 4. Gender | Was the researcher male or female? | Authors’ Information |
| 5. Experience and training | What experience or training did the researcher have? | Authors’ Information |
| Relationship with participants | | |
| 6. Relationship established | Was a relationship established prior to study commencement? | No, participants were not aware of DMN prior to study. |
| 7. Participant knowledge of the interviewer | What did the participants know about the researcher? e.g. personal goals, reasons for doing the research | Participants were aware that DMN was a PhD student and the rationale for completing the study. |
| 8. Interviewer characteristics | What characteristics were reported about the interviewer/facilitator? e.g. Bias, assumptions, reasons and interests in the research topic | Participants were aware that DMN’s background was epidemiology and that she was a PhD student at McMaster. |
| **Domain 2: study design** | | |
| Theoretical framework | | |
| 9. Methodological orientation and theory | What methodological orientation was stated to underpin the study? e.g. grounded theory, discourse analysis, ethnography, phenomenology, content analysis | Background & Study Design |
| Participant selection | | |
| 10. Sampling | How were participants selected? e.g. purposive, convenience, consecutive, snowball | Sampling and Recruitment |
| 11. Method of approach | How were participants approached? e.g. face-to-face, telephone, mail, email | Sampling and Recruitment |
| 12. Sample size | How many participants were in the study? | Characteristics of the Study Participants |
| 13. Non-participation | How many people refused to participate or dropped out? Reasons? | No patients dropped out; many patients who were approached did not participate. |
| Setting | | |
| 14. Setting of data collection | Where was the data collected? e.g. home, clinic, workplace | Data Collection |
| 15. Presence of non-participants | Was anyone else present besides the participants and researchers? | No |
| 16. Description of sample | What are the important characteristics of the sample? e.g. demographic data, date | Characteristics of the Study Participants & Table 1 |
| Data collection | | |
| 17. Interview guide | Were questions, prompts, guides provided by the authors? Was it pilot tested? | Data Collection & Appendix 3 |
| 18. Repeat interviews | Were repeat interviews carried out? If yes, how many? | No |
| 19. Audio/visual recording | Did the research use audio or visual recording to collect the data? | Data Collection |
| 20. Field notes | Were field notes made during and/or after the interview or focus group? | No field notes, but a journal was written by DMN |
| 21. Duration | What was the duration of the interviews or focus group? | Average duration was 17 minutes with a range of 14 to 24 minutes. |
| 22. Data saturation | Was data saturation discussed? | Sampling and Recruitment & Analysis |
| 23. Transcripts returned | Were transcripts returned to participants for comment and/or correction? | No, during the interview the participants were told that they could listen to their audio recordings if they chose to do so. |
| **Domain 3: analysis and findings** | | |
| Data analysis | | |
| 24. Number of data coders | How many data coders coded the data? | Analysis |
| 25. Description of the coding tree | Did authors provide a description of the coding tree? | Analysis |
| 26. Derivation of themes | Were themes identified in advance or derived from the data? | Analysis |
| 27. Software | What software, if applicable, was used to manage the data? | Data Collection |
| 28. Participant checking | Did participants provide feedback on the findings? | No |
| Reporting | | |
| 29. Quotations presented | Were participant quotations presented to illustrate the themes / findings? Was each quotation identified? e.g. participant number | Identified TDF Enablers & Identified TDF Barriers; participant numbers for each quote were not provided. |
| 30. Data and findings consistent | Was there consistency between the data presented and the findings? | Identified TDF Enablers & Identified TDF Barriers |
| 31. Clarity of major themes | Were major themes clearly presented in the findings? | Relevant TDF Domains & Table 2 |
