# Supplementary material for: Primary care provider perceptions of enablers and barriers to following guideline-recommended laboratory tests to confirm chronic kidney disease: a qualitative descriptive study
Source: BMC Fam Pract. 2018 Dec 10;19:192. doi: 10.1186/s12875-018-0879-2 (PMC6287355; doi:10.1186/s12875-018-0879-2)
Supplement: Supplementary file 3 — Interview Guide Questions. (DOCX 26 kb) [file 12875_2018_879_MOESM3_ESM.docx]

**Additional file 3: Interview Guide Questions**

| **Date and time of interview:** |  |
| --- | --- |
| **Name of interviewee:** |  |
| **Name of assistant interviewee:** |  |

*[Introducing study]*

Thank you for taking time out of your busy schedule to participate in our research study. The purpose of this study is to try and understand the factors that influence whether primary care providers follow guidelines for confirmatory laboratory tests after identifying initial abnormal kidney function to confirm if a patient has chronic kidney disease (serum creatinine, or eGFR).

Please feel free to ask us any questions about the study before we begin. Also, please remember that participation in this study is voluntary. If you would like to end the interview at any time, please do not hesitate to do so.

*[Questions about demographics and practice information]*

First, I’d like to ask you a couple questions about yourself, your practice and your patients:

| Gender |  |
| --- | --- |
| Primary care physician or nurse practitioner |  |
| May I ask your age? |  |
| How many years have you been practicing? |  |
| Did you go to medical school [the NP program] in Canada or elsewhere? And if so, where? |  |
| Rural/ urban practice location |  |
| Team or individual practice type |  |
| Approximate # of patients in practice/ rostered |  |

*[Background “nature of behaviour”]*: I’m not a clinician, but I am aware that there may be different processes by which laboratory tests are ordered in each practice. Can you please walk me through the process by which you order laboratory tests in your practice?

*[Questions based on the Theoretical Domains Framework]*

Now, I’m going to ask you some specific questions about confirmatory laboratory tests for chronic kidney disease. By this, I mean situations where you find an initial low eGFR and you order follow-up tests to confirm a diagnosis of chronic kidney disease.

There are no right or wrong answers. And some questions might seem repetitive, but these questions are based on behavioural theories to help understand which of these aspects apply to the current situation.

- K – Are you aware of guidelines for confirmatory tests for CKD?
- K – How familiar are you with these guidelines [alternative question: do you know what these guidelines recommend?]
- K – How did you hear about these guidelines? Do you think they are evidence-based?
- G – Do these guidelines conflict with other guidelines that may exist (for example, other diseases, kidney guidelines for other regions)?
- I – Do you intend/ want to order confirmatory tests for CKD?
- S – Are there any techniques or skills that you use to order confirmatory tests for CKD?
- O – Do you feel like there is value in ordering confirmatory tests for CKD?
- O – Do you generally feel positive, negative or indifferent about ordering confirmatory tests for CKD?
- Ca – How easy or difficult is it for you to regularly order confirmatory tests for CKD?
- Ca – What problems have you encountered when ordering confirmatory tests for CKD? What would help resolve these problems?
- E/R – Are there resources or environmental factors that interfere or help with ordering confirmatory tests for CKD? What are these?
- E/R – Are there competing tasks and/ or time constraints? What are these?
- G – Do you feel that you should always order confirmatory tests for CKD?
- G – Are there other things you want to do for patients’ care that might interfere with ordering these tests?
- M/A/D – Can you please describe what goes through your mind when deciding whether or not to order confirmatory tests for CKD?
- M/A/D – Might you decide not to order confirmatory tests for CKD – why or why not?
- RI – Is ordering confirmatory tests for CKD part of your professional role? Or do you think there’s another health professional who should be doing this?
- RI – Is there anything in your professional role that would help you determine whether or not to order confirmatory tests for CKD (e.g., any protocols that you follow, other technologies)?
- R – Based on previous experiences with other patients, what encourages or discourages you to order confirmatory tests for CKD?
- Co – What do you think will happen if you do not order confirmatory tests for CKD?
- Co – Do you think the benefits of ordering these tests outweigh the costs (in terms of benefits and costs for you, your patients, etc.)?
- SI - How do others (i.e. colleagues, nurses, patients, etc.) influence your opinion on whether or not to order confirmatory tests for CKD? Do patient emotions or behaviours ever influence whether or not you order confirmatory tests for CKD?
- SI - Do your colleagues generally agree with you on your views and opinions for ordering confirmatory tests for CKD?
- E – Do your emotions ever influence your decision on whether or not to order confirmatory tests for CKD (for example, cognitive overload, stress)?
- BR – Have you developed any strategies or plans to help you order confirmatory tests for CKD?

Thank you, those are all the questions we have today. Is there anything else that you wanted to add on this topic that we did not discuss today?

We are trying to recruit other physicians for our study. Do you know other healthcare providers who might be interested in participating, if so, can you please provide their name(s) and contact information?

*[Concluding interview]*

Thank you so much for participating today and making an important contribution to our study. As a reminder, we will not be using your name or any other individual-level information in our reported findings. Please feel free to contact us if you have any questions or concerns about this study. If you are interested, we can send you an overview of the findings upon completion of this study in [insert number of months]. If so, can you please provide your email address [if I do not already have it]:

| **TDF Domain Key** | |
| --- | --- |
| Knowledge | K |
| Skills | S |
| Social/ Professional Role Identity | RI |
| Beliefs about Capabilities | Ca |
| Optimism | O |
| Beliefs about Consequences | Co |
| Reinforcement | R |
| Intentions | I |
| Goals | G |
| Memory, Attention and Decision Processes | M/A/D |
| Environmental Context and Resources | E/R |
| Social Influences | SI |
| Emotion | E |
| Behavioural Regulation | BR |
